# Independent Associations of Body Composition with Dance Training Experience, Physical Activity, and Sedentary Behavior Among Polish Folk Dancers: A Cross-Sectional Study

**DOI:** 10.3390/jcm15197467

**Published:** 2026-09-25

**Authors:** Maciej Tomasz Kochman, Sylwia Baran, Wojciech Kasperek, Rafał Baran, Justyna Wyszyńska

**Affiliations:** Faculty of Health Sciences and Psychology, Medical College of Rzeszow University, 35-959 Rzeszow, Poland

**Keywords:** folk dance, body composition, physical activity, sedentary behavior, exercise, bioelectrical impedance, young adults, dance training

## Abstract

**Background**: Regular physical activity is generally associated with favorable body composition; however, little is known about the relationship between long-term folk dance participation, overall physical activity, sedentary behavior, and body composition. This study aimed to determine whether dance training experience, total physical activity, and sitting time were independently associated with BMI and body composition parameters among young adult Polish folk dancers. **Methods**: This cross-sectional study included 79 members of a Polish folk dance ensemble (51 women and 28 men) aged 18–30 years. Body composition was assessed using bioelectrical impedance analysis. Physical activity and weekday sitting time were assessed using the Polish short form of the International Physical Activity Questionnaire (IPAQ-SF), while dance experience was determined using a study-specific questionnaire. Spearman’s rank correlations and hierarchical linear regression models including sex and age as covariates were used to examine associations between dance experience, physical activity, sitting time, BMI, and body composition parameters. **Results**: Median dance experience was 30 months, while median total physical activity was 4746 MET-min/week. Dance experience was not significantly correlated with BMI or any body composition parameter. Total physical activity showed a weak positive correlation with BMI (r_s_ = 0.25, *p* = 0.025) and basal metabolic rate (r_s_ = 0.23, *p* = 0.039) but was not significantly correlated with body fat percentage, fat-free mass, muscle mass, total body water, or bone mass. In hierarchical regression models, dance experience did not significantly improve the explanation of BMI or any body composition parameter. The addition of physical activity and sitting time significantly improved the model for basal metabolic rate (ΔR^2^ = 0.028, *p* = 0.021), but not for the other outcomes. In the final models, sex was the strongest predictor of body composition, while total physical activity was independently associated with basal metabolic rate (β = 0.17, *p* = 0.007). Sitting time was not independently associated with any analyzed outcome. **Conclusions**: Among young, physically active Polish folk dancers, cumulative dance training experience was not associated with BMI or estimated body composition parameters. Sex accounted for a substantial proportion of variability in body composition, whereas total physical activity showed an independent association only with basal metabolic rate. Longitudinal studies incorporating dance-specific training volume, dietary intake, and sleep are warranted.

## 1. Introduction

Regular physical activity has well-documented beneficial effects on body composition, whilst prolonged sedentary behavior is associated with obesity, metabolic health disorders and a higher risk of chronic diseases [1,2,3]. Furthermore, a sedentary lifestyle has been identified as an independent risk factor for adverse health outcomes and may undermine the beneficial effects of physical activity, even among those who achieve the recommended levels of physical activity [4]. These findings highlight the importance of investigating the association between a sedentary lifestyle and body composition, including in populations that are otherwise physically active.

Body composition parameters are important indicators for assessing the effects of physical activity, as well as physical inactivity, in adult populations [5,6]. Body composition comprises several components, including fat mass and fat-free mass, the latter of which comprises skeletal muscle mass, total body water, and bone mass [7]. The relative proportions of these components vary depending on the type, duration and intensity of the physical activity performed [8]. Therefore, investigating the effects of various forms of physical activity on body composition is of particular importance. Previous studies have shown that resistance training reduces body fat percentage, fat mass and visceral fat mass [8,9,10]. Similarly, a meta-analysis conducted by Jayedi et al. found that 30 min of aerobic exercise promotes a reduction in body weight and waist circumference in overweight individuals, whilst achieving clinically significant improvement requires at least 150 min of aerobic exercise per week [11].

Due to its specific characteristics, including complex movement patterns and expressive choreography, folk dancing can combine aerobic demands with repeated muscular loading and dynamic movements. This is supported by research carried out among young folk dancers, whose regular dance training led to improvements in aerobic capacity and muscle strength [12]. Similarly, Maciejczyk and Feć demonstrated that folk dancing is a physically demanding activity, and that folk dancers exhibit high levels of physical fitness [13]. Our previous research has focused primarily on the biomechanics of movement and the impact of dance training on pain; however, we also observed a positive significant correlation between experience of folk dance training and BMI in folk dancers [14,15]. A review of the available literature indicates that relatively few studies have examined the relationships between experience of folk dance training, overall levels of physical activity and body composition parameters. Despite the growing number of scientific publications devoted to folk dance and the increasing interest in cultural heritage and the educational aspects associated with this form of physical activity [16,17,18], little is known about the relationship between long-term folk dance participation and body composition. Filling this research gap may be significant from a scientific perspective and also provide a basis for drawing practical conclusions. A better understanding of the relationship may support the development of strategies for monitoring health status and body composition parameters in people who engage in regular physical activity. This aspect seems particularly important, as a high level of physical activity does not preclude a sedentary lifestyle and its potentially adverse effects on health.

The aim of this study was to determine whether dance experience, total physical activity, and sitting time were independently associated with BMI and body composition among adult folk dancers, and whether physical activity and sitting time provided additional explanatory value beyond sex, age, and dance experience. Based on this aim, four hypotheses were formulated: H1: Longer dance training experience is associated with BMI, body composition parameters, and BMR. H2: Higher total physical activity is associated with BMI, body composition parameters, and BMR. H3: Greater weekday sitting time is associated with BMI, body composition parameters, and BMR. H4: Total physical activity and weekday sitting time provide additional explanatory value for BMI and body composition parameters beyond sex, age, and dance training experience.

## 2. Materials and Methods

### 2.1. Study Design and Study Sample

This observational, cross-sectional study was conducted among members of the Połoniny Song and Dance Ensemble in Rzeszów, Poland, between 2024 and 2025. Participants were recruited from among dancers engaged in the regular activities of the ensemble during the data collection period. Individuals who were available during the scheduled assessment sessions were screened for eligibility. Those who met the eligibility criteria and provided written informed consent were enrolled in the study.

The inclusion criteria were as follows: (1) age between 18 and 30 years; (2) active membership in the *Połoniny* Song and Dance Ensemble; (3) regular participation in dance training; (4) absence of any current health condition that could prevent participation in the assessments; (5) absence of contraindications to bioelectrical impedance analysis; and (6) provision of written informed consent.

The exclusion criteria comprised: (1) a current musculoskeletal injury, fracture, or other condition preventing safe participation in the study procedures; (2) pregnancy; (3) the presence of an implanted electronic medical device; (4) any other contraindication to bioelectrical impedance analysis; and (5) incomplete questionnaire or body composition data.

All active members of the ensemble were invited to participate in the study. Of the 82 individuals who initially agreed to participate, 3 were not included in the final sample because they were either absent on the scheduled assessment day or exceeded the upper age limit of 30 years. Consequently, the final analytical sample comprised 79 dancers, including 51 women (64.6%) and 28 men (35.4%), with a mean age of 22.3 ± 3.13 years. All participants included in the study completed both the questionnaire and body composition assessment and were included in the statistical analyses. The recruitment and selection process is illustrated in Figure 1.

A sensitivity analysis was conducted using G*Power version 3.1.9.7 for a fixed-effects multiple regression model testing the increase in R^2^ attributable to the addition of two predictors—total physical activity and sitting time—to a model containing five predictors in total. With a sample size of *n* = 79, a significance level of α = 0.05, and statistical power of 0.80, the minimum detectable incremental effect size was f^2^ = 0.127. For comparison, detecting a medium incremental effect of f^2^ = 0.15 under the same assumptions required a minimum sample size of 68 participants. Thus, the available sample was adequately powered to detect medium-sized incremental effects, whereas smaller effects may have remained undetected.

### 2.2. Anthropometric Measurements

All anthropometric and body composition measurements were performed in the morning, following an overnight fast of at least 8 h. The assessments were conducted by the same trained investigator under standardized conditions. The measuring devices were calibrated before data collection and checked in accordance with the manufacturers’ recommendations.

Standing height was measured to the nearest 0.1 cm using a Tanita HR-200 stadiometer (Tanita Corporation, Tokyo, Japan). Participants were assessed without footwear, standing upright with their heels together and their head positioned in the Frankfort horizontal plane. Stadiometer-based standing height measurement has demonstrated excellent intra- and inter-rater reliability, with reported ICC values of 0.99–1.00 [19].

Body weight was measured to the nearest 0.1 kg using a calibrated Tanita BC-420MA body composition analyzer (Tanita Corporation, Tokyo, Japan). During the measurement, participants were barefoot and wore light indoor clothing. Body mass index (BMI) was calculated as body weight in kilograms divided by height in meters squared and expressed in kg/m^2^ [20].

### 2.3. Body Composition Assessment

Body composition was assessed using bioelectrical impedance analysis with a Tanita BC-420MA body composition analyzer (Tanita Corporation, Tokyo, Japan). This method estimates body composition by measuring the impedance of body tissues to a low-intensity alternating electrical current. BIA is considered a valid and highly reliable method for estimating body fat percentage in adults, with previous research demonstrating strong agreement with DXA (R^2^ = 0.92) and excellent test–retest reliability, with repeated measurements differing by less than 0.2% [21].

Participants stood barefoot and motionless on the analyzer platform, ensuring full contact between the soles of both feet and the stainless-steel electrodes. Before each measurement, the participant’s sex, age, body height, and body-type setting were entered into the analyzer. All measurements were performed in the morning after an overnight fast of at least 8 h to standardize the assessment conditions and minimize variability associated with recent food and fluid intake [22]. The body composition variables included in the statistical analyses were BFP (%), FFM (%), muscle mass (%), TBW (%), and estimated bone mass (%). Basal metabolic rate was expressed in kilocalories per day (kcal/day).

### 2.4. Physical Activity and Dance Experience

Physical activity was assessed using the officially approved Polish version of the short form of the International Physical Activity Questionnaire (IPAQ-SF), referring to the preceding 7 days. The IPAQ-SF comprises seven items assessing the frequency and duration of vigorous-intensity physical activity, moderate-intensity physical activity, walking, and time spent sitting. It captures physical activity undertaken across different domains of daily life, including occupational, transport-related, household, and leisure-time activities; however, the short form does not provide separate domain-specific scores. The IPAQ demonstrated good test–retest reliability (Spearman’s ρ approximately 0.80) and acceptable criterion validity against accelerometry (median ρ approximately 0.30) in the original 12-country validation study [23].

Participants reported the number of days during the preceding 7 days on which they performed vigorous-intensity activity, moderate-intensity activity, and walking, as well as the average duration of each type of activity on one of those days. In accordance with the IPAQ methodology, only physical activities performed continuously for at least 10 min were included. Vigorous-intensity activity was defined as activity causing very rapid breathing and a substantial increase in heart rate, whereas moderate-intensity activity was defined as activity causing a moderate increase in breathing and heart rate.

Physical activity was expressed in metabolic equivalent task minutes per week (MET-min/week). The following MET values recommended for the short form of the IPAQ were applied: 3.3 METs for walking, 4.0 METs for moderate-intensity activity, and 8.0 METs for vigorous-intensity activity. For each intensity category, MET-min/week were calculated by multiplying the assigned MET value by the number of days per week and the reported duration in minutes per day. Total physical activity was calculated as the sum of walking, moderate-intensity activity, and vigorous-intensity activity and was treated as a continuous variable in the statistical analyses.

Sitting time was assessed using the final item of the Polish IPAQ-SF. Participants reported their usual daily sitting time on weekdays during the preceding 7 days, including sitting at home, work or school, in motor vehicles, and while watching television. Sitting time was analyzed separately from total physical activity, multiplied by five, and expressed as total weekday sitting time in min/week [24].

Dance experience was assessed using a study-specific questionnaire and was defined as the cumulative duration of regular dance training, excluding periods during which participants did not participate in training. Dance experience was expressed in months in the descriptive analyses and converted to years for the regression analyses.

In addition to cumulative dance experience, participants reported their usual weekly time devoted to dance training, training frequency, and the typical duration of a single training session. These variables were used to provide a more detailed descriptive characterization of dance-specific training exposure. Dance-specific training intensity was not assessed directly.

### 2.5. Ethical Considerations

The study protocol was reviewed and approved by the Bioethics Committee of the University of Rzeszów, Poland (Resolution No. 013/04/2024, dated 10 April 2024). The study was conducted in accordance with the principles of the Declaration of Helsinki and applicable national and institutional regulations governing research involving human participants.

Before enrolment, all potential participants received detailed information regarding the purpose, scope, and procedures of the study. They were informed that participation was entirely voluntary, that the collected data would be used exclusively for scientific purposes, and that they could withdraw from the study at any stage without providing a reason and without any adverse consequences.

Written informed consent was obtained from each participant before the commencement of any study procedures. All participants were adults and provided written consent both to participate and to allow the collected data and examination results to be used for scientific purposes. Participant confidentiality was maintained throughout the study, and the data were analyzed in an anonymized form that did not allow individual participants to be identified.

### 2.6. Statistical Analysis

Statistical analyses were performed using Statistica 13.3. Continuous variables were summarized using the mean, median, minimum and maximum values, lower and upper quartiles, and standard deviation.

Dance experience was presented in months in the descriptive analyses and converted to years for the regression analyses to facilitate interpretation of the unstandardized regression coefficients. Total physical activity was calculated as the sum of vigorous-intensity activity, moderate-intensity activity, and walking and expressed in MET-min/week. Because of its positively skewed distribution, total physical activity was natural-log transformed before inclusion in the regression models. Sitting time was analyzed in min/week. To improve interpretability, unstandardized coefficients for sitting time were presented per 1000 min/week increase.

Spearman’s rank correlation coefficients were used to examine associations between dance experience, total physical activity, sitting time, BMI, and body composition parameters because several variables, particularly dance experience and physical activity, were not normally distributed.

Hierarchical linear regression was used to assess the independent associations of sex, age, dance experience, total physical activity, and sitting time with BMI and body composition. Sex and age were included a priori as covariates in all regression models to account for their potential confounding effects on BMI and body composition. Three sequential models were constructed for each outcome. Model 1 included sex and age; Model 2 additionally included dance experience; and Model 3 further included the natural logarithm of total physical activity and sitting time. This approach allowed assessment of the additional explanatory contribution of dance experience beyond sex and age, as well as the incremental contribution of physical activity and sitting time.

BMI was analyzed as a separate outcome and was not included as a predictor in models of body composition. Separate regression models were constructed for body fat percentage, fat-free mass percentage, muscle mass percentage, total body water percentage, estimated bone mass percentage, and basal metabolic rate.

Regression results were reported as unstandardized coefficients (B), standard errors (SE), standardized coefficients (β), 95% confidence intervals, and *p* values. Model fit was evaluated using R^2^, adjusted R^2^, and the F statistic. Changes between consecutive hierarchical models were assessed using ΔR^2^, ΔF, and the corresponding *p* value. Assumptions of the final regression models were assessed prior to interpretation. Multicollinearity was evaluated using variance inflation factors (VIF) and tolerance values. The distribution of residuals was assessed using the Shapiro–Wilk test, while homoscedasticity was evaluated using the Breusch–Pagan test. Potentially influential observations were assessed using externally studentized residuals, leverage values, and Cook’s distance. VIF values below 5 and tolerance values above 0.20 were considered indicative of the absence of problematic multicollinearity. Statistical significance was set at *p* < 0.05. No formal adjustment for multiple comparisons was applied; therefore, nominally significant findings were interpreted cautiously in view of the number of correlations and regression models examined.

## 3. Results

The study included 79 folk dancers with a mean age of 22.3 ± 3.13 years. The participants’ dance experience varied considerably (median: 30 months; range: 3–204 months). Participants reported a mean weekly dance training time of 3.1 ± 1.50 h/week (median: 3.0 h/week; range: 1–8 h/week). Most participants (44.3%) attended dance training twice per week, followed by those training once per week (34.2%), three times per week (19.0%), and more than three times per week (2.5%). The most commonly reported duration of a single training session was 60–90 min (84.8%), followed by 90–120 min (10.1%), 30–60 min (3.8%), and more than 120 min (1.3%).

The mean BMI was 22.7 kg/m^2^. Physical activity levels showed considerable variability (median: 4746 MET-min/week), as did the amount of time spent sitting. Detailed characteristics of the study group are presented in Table 1.

The correlation analysis revealed no significant associations between dance experience and BMI, or any of the body composition parameters analyzed (*p* > 0.05). Overall physical activity levels showed a weak but statistically significant positive correlation with BMI (r_s_ = 0.25; *p* = 0.025) and basal metabolic rate (BMR) (r_s_ = 0.23; *p* = 0.039). No significant associations were observed between the level of physical activity and body fat percentage, fat-free mass (FFM), muscle mass, body water content or bone mass. Time spent sitting was not significantly associated with any of the parameters analyzed. Detailed results are presented in Table 2.

Regression analysis showed that the model including sex and age significantly explained 24.1% of the variance in BMI (*p* < 0.001). The addition of dance experience did not significantly improve the predictive value of the model (*p* = 0.437). Extending the model to include total physical activity and weekday sitting time increased the explained variance to 28.1%; however, this change was not statistically significant (*p* = 0.185). Detailed results are presented in Table 3.

In the first regression model, sex was a significant predictor of BMI. Men had higher BMI values than women (β = 0.44; *p* < 0.001), whilst age was not significantly associated with BMI. After including dance experience in the model, its effect remained statistically insignificant (B = −0.05; β = −0.08; *p* = 0.437). In the final model, sex remained a significant predictor of BMI (B = 2.49; β = 0.40; *p* < 0.001). Dance experience was not significantly associated with BMI (β = −0.09; *p* = 0.357). Total physical activity showed a positive, but statistically non-significant, association with BMI (β = 0.19; *p* = 0.068), whereas weekday sitting time was not significantly associated with BMI (β = 0.03; *p* = 0.733). Detailed results are presented in Table 4.

All basic regression models, including sex and age, were statistically significant and explained between 24.7% (bone mass) and 38.5% (muscle mass) of the variance in the analyzed parameters. The best model fit was observed for BMR, for which the basic model explained 71.7% of its variability. The inclusion of a variable relating to dance experience did not result in a statistically significant improvement in any of the models (*p* > 0.05). Extending the models to include physical activity and time spent sitting increased the R^2^ values; however, a significant improvement was observed only for BMR (ΔR^2^ = 0.028; ΔF(2.73) = 4.05; *p* = 0.021). Detailed results are presented in Table 5.

After adjusting for sex and age, dance experience showed no significant association with any of the analyzed body composition parameters or with BMR. In Model 2, the standardized regression coefficients were low (−0.08 ≤ β ≤ 0.10). Additional adjustment for total physical activity and weekday sitting time did not materially alter the regression coefficients for dance experience. In the final model, none of the coefficients relating to dance experience reached statistical significance, and all confidence intervals included zero. Detailed data are shown in Table 6.

In the final regression models adjusted for age, dance experience, total physical activity, and weekday sitting time, sex was significantly associated with all analyzed body composition parameters and BMR. Compared with women, men had a lower body fat percentage and higher FFM, muscle mass, TBW, bone mass, and BMR. The strongest association was observed for BMR (β = 0.82; *p* < 0.001). Age was significantly associated only with TBW, with older age being associated with lower body water content (β = −0.32; *p* = 0.004). Dance experience was not a significant predictor of any of the analyzed parameters. Physical activity was significantly associated only with BMR, with higher levels of activity corresponding to higher basal metabolic rate values (β = 0.17; *p* = 0.007). Time spent sitting showed no significant independent associations with the analyzed variables. Detailed data are presented in Table 7. Diagnostic assessment of the final regression models indicated no problematic multicollinearity (VIF: 1.04–1.22; tolerance: 0.82–0.96), significant departures from residual normality (all Shapiro–Wilk *p* ≥0.126), or significant heteroscedasticity (all Breusch–Pagan *p* ≥0.059). No observation showed excessive influence based on Cook’s distance (maximum = 0.238) or leverage (maximum = 0.193).

## 4. Discussion

In this study, we investigated whether dance training experience was independently associated with body composition parameters among young adult Polish folk dancers after controlling for sex, age, overall physical activity and sedentary behavior. Our findings showed that, contrary to our hypothesis, dance experience was not independently associated with any analyzed body composition parameter after adjusting for sex, age, physical activity level and sitting time. We hypothesized that longer dance training experience would be associated with lower body fat percentage, greater fat-free mass and greater muscle mass; however, this hypothesis was not confirmed. These findings indicate that, within the present sample, we did not observe a significant independent association between longer folk dance training and body composition. This result should not, however, be interpreted as evidence that no relationship exists. The relatively homogeneous and physically active sample, the cross-sectional design, and the use of cumulative dance duration as the primary indicator of training exposure may have limited our ability to detect weaker or more specific associations.

The absence of correlations between dance experience and body composition was further confirmed by regression analyses. The lack of association between dance experience and body composition may indicate that the duration of dance participation alone is insufficient to determine body composition differences. This finding may be explained by the relatively homogeneous lifestyle of participants, or the influence of other determinants such as diet and genetic predisposition. This is, however, contrary to the previous research, as Malkogeorgos et al. demonstrated that participating in three 60 min folk dance sessions per week led to weight loss and beneficial changes in body composition among adults who had previously been physically inactive [25].

An important consideration when interpreting these findings is that cumulative duration of dance participation may not fully reflect the actual dose of dance training. Recent research among female collegiate dancers similarly characterized dance exposure using multiple components, including dance-specific course load, the duration and frequency of dance classes, rehearsal time, and performance-related activities, rather than relying on a single measure of dance history [26]. This is consistent with the view that cumulative dance experience alone may not fully capture actual training exposure. Although participants in the present study reported their weekly dance-training time, training frequency, and typical session duration, substantial variation was observed in weekly training frequency and volume. Moreover, dance-specific training intensity was not assessed directly. Consequently, cumulative training history alone may not have adequately captured differences in weekly training load between dancers. Another limitation is the variability in dance-training frequency and weekly training volume within the sample. Although these parameters were recorded and described, they were not included as independent predictors in the regression models, and dance-specific training intensity was not directly assessed. Future studies should therefore consider more comprehensive measures of dance exposure, including weekly training volume, frequency, duration, intensity, and, where possible, objectively monitored training load. It would also be beneficial to stratify dancers according to weekly training frequency or total weekly training volume and to incorporate more detailed or objective measures of dance-specific training load.

Our findings also contrast with results from studies conducted among ballet dancers, ballroom dancers, and dancesport athletes, which have reported discipline-specific anthropometric and body composition characteristics [27,28,29,30]. In competitive dancing, lean physique, specific body proportions and aesthetic presentation are essential components of performance requirements, which may also contribute to increased pressure to maintain a low body weight and lean physique [31,32,33]. Competitive dancers, especially females, have been reported in some studies to exhibit lower body fat compared with healthy controls [32]. These differences may reflect training-related adaptations, as well as sport-specific selection criteria and aesthetic demands [31,32]. Moreover, aesthetic pressures have also been associated with a higher prevalence of eating psychopathology among ballet dancers [33].

In our study, higher total physical activity level was weakly but positively correlated with BMI. This means that individuals with higher declared physical activity levels were characterized by slightly higher BMI values on average. A similar relationship was observed for BMR. Although total physical activity was independently associated with BMR in the final regression model, the standardized effect was relatively small (β = 0.17), and the practical or physiological significance of this association should therefore be interpreted cautiously. However, physical activity was not significantly associated with body fat percentage, FFM, muscle mass, total body water or bone mass, which is an interesting finding in relation to higher BMI values. As is well known, BMI does not distinguish between fat mass and fat-free mass [34,35]. Therefore, higher BMI values in physically active individuals may partly reflect greater fat-free mass rather than increased adiposity [34]. These results are partially supported by the literature, as higher levels of physical activity are generally associated with increased energy expenditure and lower obesity risk [36]. In already active populations, differences in habitual physical activity levels may have a limited impact on total energy expenditure and body composition due to physiological adaptations [37].

Contrary to our expectations, sedentary behavior was not independently associated with body composition and BMI. One possible explanation is that the relatively high level of physical activity in this sample may have reduced the variability in the potential contribution of sedentary behavior to body composition. However, this interpretation remains speculative and cannot be confirmed by the present cross-sectional data. In addition, the relatively limited variability in body composition and the use of self-reported sitting time may have reduced the ability to detect weaker associations.

In summary, dance experience did not provide additional explanatory value beyond sex and age. Importantly, adjustment for physical activity and sedentary behavior did not alter the relationship between dance experience and body composition, indicating that the absence of association was not explained by differences in lifestyle behaviors.

Sex emerged as the strongest determinant of body composition in our study. Men exhibited higher values of fat-free mass, muscle mass, total body water, bone mass and BMR, whereas women had a higher percentage of body fat. These differences are consistent with commonly known sex differences in body composition and hormonal regulation in the general population [38,39].

This study provides novel insight into body composition determinants among folk dancers, a population that remains underrepresented in the scientific literature, especially Polish folk dancers. We also used validated assessment tools, including IPAQ and bioelectrical impedance analysis, which strengthens the reliability of the obtained measurements. The sample size of 79 participants represents a relatively large cohort considering the limited availability of research involving Polish folk dancers. In addition, this sample size compares favorably with previous studies conducted among dancer populations.

Despite these strengths, several limitations should be acknowledged. The cross-sectional design limits causal interpretations of the observed associations. Moreover, although relevant lifestyle factors were included, other determinants of body composition, such as diet and sleep, were not assessed. In addition, although the IPAQ-SF captures physical activity performed across different domains of daily life, non-dance physical activity was not monitored separately or objectively. Furthermore, the IPAQ-SF relies on self-reported information regarding physical activity performed during the preceding seven days and may therefore be subject to recall and reporting bias. This limitation may be particularly relevant in physically active populations, such as dancers, who may have difficulty accurately estimating the duration and intensity of their habitual activities. Consequently, some measurement error in total physical activity cannot be excluded and may have attenuated or distorted the observed associations. Another limitation concerns the use of bioelectrical impedance analysis to assess body composition. Although BIA is a practical and widely used method and the assessment conditions were standardized in the present study, the technique provides estimated rather than direct measurements of body composition. Its estimates may be influenced by factors such as hydration status and other physiological conditions. The standardized morning measurements following an overnight fast were intended to reduce such variability; however, measurement error inherent to BIA cannot be completely excluded. In addition, the studied population consisted of young and relatively homogeneous participants, which may limit the variability of body composition parameters and reduce the ability to detect weaker associations. Moreover, multiple correlations and regression models were examined across several related outcomes, which increases the possibility of type I error. As no formal correction for multiple testing was applied, isolated nominally significant findings should therefore be interpreted with caution.

Overall, our findings suggest that in young, physically active folk dancers, duration of dance participation alone may not be a sufficient predictor of differences in body composition. Instead, individual characteristics such as sex and broader lifestyle factors may play a more substantial role in explaining these associations.

From a practical perspective, body composition monitoring in young folk dancers should consider sex and overall physical activity rather than cumulative dance experience or sitting time alone. Dance-specific training volume and intensity may also be useful when evaluating individual training exposure and health status.

## 5. Conclusions

In young adult Polish folk dancers, dance training experience was not independently associated with BMI or body composition parameters after adjustment for sex, age, total physical activity, and sedentary time. These findings suggest that within a physically active and relatively homogeneous group, the duration of participation in folk dance alone may not explain differences in body composition.

Sex was the strongest determinant of body composition, with men showing higher fat-free mass, muscle mass, total body water, bone mass, and basal metabolic rate, while women presented higher body fat percentage. Total physical activity was independently associated only with basal metabolic rate, whereas sedentary time was not related to the analyzed body composition parameters.

Further longitudinal studies, including additional lifestyle factors such as dietary intake and sleep patterns, are needed to determine whether long-term folk dance participation contributes to changes in body composition. Future research should consider more homogeneous age groups and, where appropriate, a narrower age range to reduce age-related variability, as well as include more detailed measures of dance-specific training exposure, including weekly training volume, training frequency, and intensity.

## Figures and Tables

**Figure 1:**
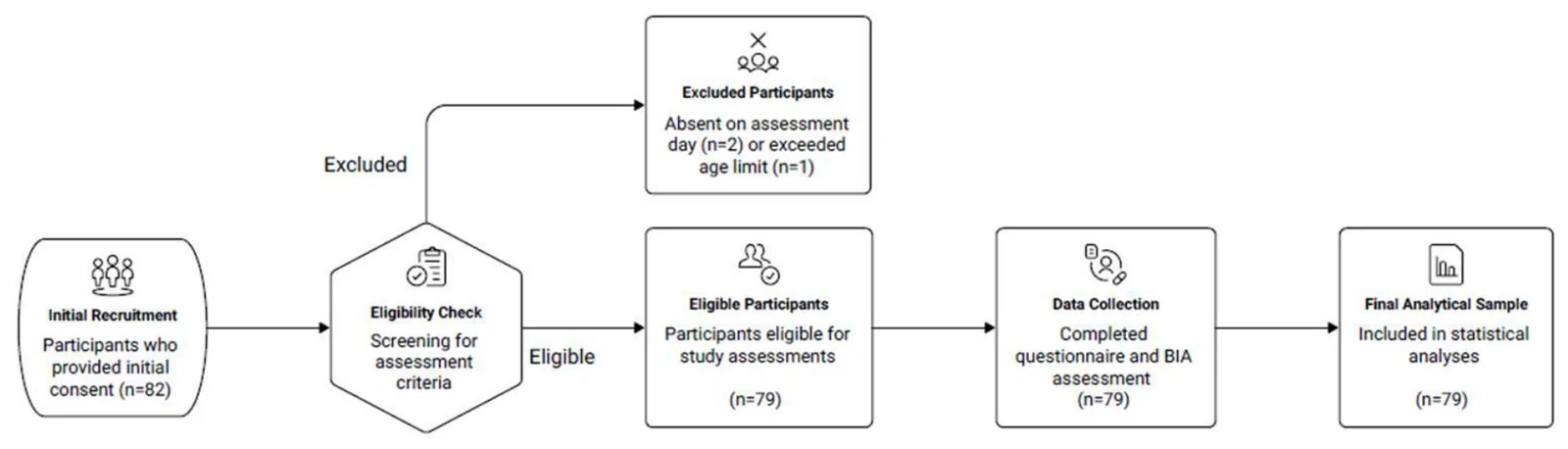
Recruitment and selection process of study participants.

**Table 1:** Characteristics of the Study Group, BMI, Body Composition Parameters, Total Physical Activity, and Weekday Sitting Time.

Variable	Descriptive Statistics							
	*n*	M	Me	Min.	Max.	Q1	Q3	SD
Age (years)	79	22.3	21.0	18.0	30.0	20.0	24.0	3.13
Dance experience (months)	79	50.6	30.0	3.0	204.0	12.0	72.0	53.03
Weekly dance training time (h/week)	79	3.1	3.0	1.0	8.0	2.0	4.0	1.50
BMI (kg/m^2^)	79	22.7	22.9	16.7	29.9	20.5	25.2	2.98
BFP (%)	79	19.9	19.8	8.2	35.0	14.9	24.1	6.12
FFM (%)	79	80.1	80.1	65.0	91.8	75.9	85.2	6.12
Muscle mass (%)	79	76.0	76.1	61.8	87.2	72.1	80.8	5.83
TBW (%)	79	56.0	56.2	45.9	66.9	52.9	59.4	4.64
Bone mass (%)	79	4.0	4.0	3.3	5.0	3.8	4.3	0.31
BMR (kcal/day)	79	1622.2	1496.0	1202.0	2409.0	1381.0	1903.0	320.11
Total physical activity (MET-min/week)	79	6858.0	4746.0	876.0	24360.0	2748.0	9630.0	5635.25
Weekday sitting time (min/week)	79	2137.2	2100.0	180.0	5400.0	1200.0	3000.0	1163.61

*N*—number of participants; M—mean; Me—median; Min.—minimum; Max.—maximum; Q1—lower quartile; Q3—upper quartile; SD—standard deviation; BMI—body mass index; BFP–body fat percentage; FFM—fat-free mass; TBW—total body water; BMR—basal metabolic rate; MET-min/week—metabolic equivalent task minutes per week.

**Table 2:** Spearman Rank Correlations of Dance Training Experience, Total Physical Activity, and Weekday Sitting Time with BMI and Body Composition Parameters.

Variable	Dance Experience (Months)	Total Physical Activity (MET-Min/Week)	Weekday Sitting Time (Min/Week)
BMI (kg/m^2^)	*r_s_* = 0.03 *p* = 0.780	*r_s_* = 0.25 *p* = 0.025	*r_s_* = 0.01 *p* = 0.910
BFP (%)	*r_s_* = −0.11 *p* = 0.350	*r_s_* = 0.03 *p* = 0.777	*r_s_* = 0.04 *p* = 0.708
FFM (%)	*r_s_* = 0.11 *p* = 0.344	*r_s_* = −0.04 *p* = 0.757	*r_s_* = −0.04 *p* = 0.720
Muscle Mass (%)	*r_s_* = 0.11 *p* = 0.352	*r_s_* = −0.03 *p* = 0.779	*r_s_* = −0.04 *p* = 0.719
TBW (%)	*r_s_* = 0.05 *p* = 0.686	*r_s_* = −0.07 *p* = 0.550	*r_s_* = 0.01 *p* = 0.965
Bone Mass (%)	*r_s_* = 0.09 *p* = 0.407	*r_s_* = −0.07 *p* = 0.540	*r_s_* = −0.06 *p* = 0.570
BMR (kcal/day)	*r_s_* = −0.04 *p* = 0.729	*r_s_* = 0.23 *p* = 0.039	*r_s_* = −0.07 *p* = 0.546

r_s_—Spearman rank correlation coefficient; *p*—*p* value; BMI—body mass index; BFP—body fat percentage; FFM—fat-free mass; TBW—total body water; BMR—basal metabolic rate; MET-min/week—metabolic equivalent task minutes per week.

**Table 3:** Fit of Hierarchical Linear Regression Models for BMI.

Model	Variables Included in the Model	R^2^	Adj. R^2^	F (df1, df2)	*p* for Model	ΔR^2^	ΔF (df1, df2)	*p* for Δ
1	Sex, Age	0.241	0.221	12.04 (2. 76)	<0.001	**-**	-	-
2	Sex, Age, Dance experience	0.247	0.217	8.19 (3. 75)	<0.001	0.006	0.61 (1. 75)	0.437
3	Sex, Age, Dance experience, Total physical activity, Weekday sitting time	0.281	0.232	5.70 (5. 73)	<0.001	0.034	1.73 (2. 73)	0.185

R^2^—coefficient of determination; Adj. R^2^—adjusted coefficient of determination; F—F-statistic assessing the significance of the overall regression model; df_1_, df_2_—degrees of freedom; ΔR^2^—change in the coefficient of determination compared with the immediately preceding model; ΔF—change in the F-statistic; *p* for Δ—*p* value for the change in model fit.

**Table 4:** Coefficients of Hierarchical Linear Regression Models for BMI.

Model	Predictor	B [95% CI]	SE	β	*P*
1	Sex: Male (ref. Female)	2.74 [1.43; 4.06]	0.66	0.44	<0.001
	Age (years)	0.10 [−0.10; 0.30]	0.10	0.11	0.320
2	Sex: Male (ref. Female)	2.70 [1.38; 4.02]	0.66	0.44	<0.001
	Age (years)	0.12 [−0.09; 0.33]	0.10	0.13	0.254
	Dance experience (years)	−0.05 [−0.19; 0.08]	0.07	−0.08	0.437
3	Sex: Male (ref. Female)	2.49 [1.14; 3.84]	0.68	0.40	<0.001
	Age (years)	0.15 [−0.06; 0.35]	0.10	0.15	0.168
	Dance experience (years)	−0.06 [−0.20; 0.07]	0.07	−0.09	0.357
	Natural log of total physical activity [MET-min/week]	0.71 [−0.05; 1.47]	0.38	0.19	0.068
	Weekday sitting time [min/week]	0.09 [−0.43; 0.61]	0.26	0.03	0.733

B—unstandardized regression coefficient; SE—standard error; β—standardized regression coefficient; CI—95% confidence interval; *p*—*p* value. Female sex was used as the reference category. Dance experience was expressed in years. Total physical activity was natural-log transformed before inclusion in Model 3. For weekday sitting time, B, SE, and 95% CI are presented per 1000 min/week increase.

**Table 5:** Fit of Hierarchical Regression Models for Body Composition Parameters and BMR.

Variable	Model	R^2^	Adj. R^2^	F (df1, df2)	*p* for Model	ΔR^2^	ΔF (df1, df2)	*p* for Δ
BFP (%)	1	0.379	0.363	23.20 (2. 76)	<0.001	—	—	—
	2	0.385	0.361	15.66 (3. 75)	<0.001	0.006	0.74 (1. 75)	0.393
	3	0.421	0.381	10.60 (5. 73)	<0.001	0.036	2.25 (2. 73)	0.113
FFM (%)	1	0.378	0.362	23.12 (2. 76)	<0.001	—	—	—
	2	0.384	0.360	15.60 (3. 75)	<0.001	0.006	0.72 (1. 75)	0.397
	3	0.420	0.380	10.56 (5. 73)	<0.001	0.036	2.24 (2. 73)	0.114
Muscle Mass (%)	1	0.385	0.369	23.81 (2. 76)	<0.001	—	—	—
	2	0.391	0.367	16.06 (3. 75)	<0.001	0.006	0.72 (1. 75)	0.398
	3	0.427	0.387	10.86 (5. 73)	<0.001	0.035	2.25 (2. 73)	0.112
TBW (%)	1	0.264	0.245	13.66 (2. 76)	<0.001	—	—	—
	2	0.275	0.246	9.47 (3. 75)	<0.001	0.010	1.07 (1. 75)	0.304
	3	0.317	0.270	6.76 (5. 73)	<0.001	0.042	2.23 (2. 73)	0.115
Bone Mass (%)	1	0.247	0.227	12.44 (2. 76)	<0.001	—	—	—
	2	0.255	0.225	8.55 (3. 75)	<0.001	0.008	0.83 (1. 75)	0.365
	3	0.290	0.242	5.98 (5. 73)	<0.001	0.036	1.83 (2. 73)	0.167
BMR (kcal/day)	1	0.717	0.710	96.45 (2. 76)	<0.001	—	—	—
	2	0.717	0.706	63.47 (3. 75)	<0.001	<0.001	0.01 (1. 75)	0.916
	3	0.746	0.728	42.80 (5. 73)	<0.001	0.028	4.05 (2. 73)	0.021

R^2^—coefficient of determination; adj. R^2^—adjusted coefficient of determination; F—F statistic for the overall model; df1, df2—degrees of freedom; ΔR^2^—change in the coefficient of determination relative to the immediately preceding model; ΔF—F statistic for the change in model fit; *p* for Δ—*p* value for the change in model fit. Model 1 included sex and age; Model 2 additionally included dance experience; Model 3 additionally included the natural logarithm of total physical activity and weekday sitting time.

**Table 6:** Regression Coefficients for Dance Experience in Models of Body Composition Parameters and BMR Before and After Additional Adjustment for Total Physical Activity and Weekday Sitting Time.

Outcome	Model	B [95% CI]	SE	β	*P*
BFP (%)	2	−0.11 [−0.37; 0.15]	0.13	−0.08	0.393
	3	−0.14 [−0.39; 0.12]	0.13	−0.10	0.282
FFM (%)	2	0.11 [−0.15; 0.37]	0.13	0.08	0.397
	3	0.14 [−0.12; 0.39]	0.13	0.10	0.285
Muscle Mass (%)	2	0.10 [−0.14; 0.35]	0.12	0.08	0.398
	3	0.13 [−0.11; 0.37]	0.12	0.10	0.285
TBW (%)	2	0.11 [−0.10; 0.32]	0.11	0.10	0.304
	3	0.13 [−0.08; 0.34]	0.10	0.13	0.211
Bone Mass (%)	2	0.007 [−0.008; 0.021]	0.007	0.09	0.365
	3	0.008 [−0.006; 0.022]	0.007	0.11	0.276
BMR (kcal/day)	2	−0.48 [−9.57; 8.61]	4.56	−0.01	0.916
	3	−1.27 [−10.06; 7.52]	4.41	−0.02	0.774

B—unstandardized regression coefficient; SE—standard error; β—standardized regression coefficient; CI—95% confidence interval; *p*—*p* value. Model 2 included sex, age, and dance experience. Model 3 additionally included the natural logarithm of total physical activity and weekday sitting time. The B coefficient represents the change in the outcome associated with each additional year of dance experience.

**Table 7:** Coefficients of the Final Regression Models for Body Composition Parameters and BMR.

Outcome	Predictor	B [95% CI]	SE	β	*P*
BFP (%)	Sex: Male (ref. Female)	−8.93 [−11.42; −6.44]	1.25	−0.70	<0.001
	Age (years)	0.32 [−0.07; 0.70]	0.19	0.16	0.105
	Dance experience (years)	−0.14 [−0.39; 0.12]	0.13	−0.10	0.282
	Natural log of total physical activity (MET-min/week)	1.23 [−0.17; 2.63]	0.70	0.16	0.085
	Weekday sitting time (min/week)	−0.48 [−1.43; 0.47]	0.48	−0.09	0.319
FFM (%)	Sex: Male (ref. Female)	8.92 [6.43; 11.41]	1.25	0.70	<0.001
	Age (years)	−0.32 [−0.70; 0.07]	0.19	−0.16	0.105
	Dance experience (years)	0.14 [−0.12; 0.39]	0.13	0.10	0.285
	Natural log of total physical activity (MET-min/week)	−1.23 [−2.63; 0.18]	0.70	−0.16	0.085
	Weekday sitting time (min/week)	0.48 [−0.48; 1.43]	0.48	0.09	0.323
Muscle Mass (%)	Sex: Male (ref. Female)	8.56 [6.21; 10.92]	1.18	0.71	<0.001
	Age (years)	−0.30 [−0.66; 0.06]	0.18	−0.16	0.104
	Dance experience (years)	0.13 [−0.11; 0.37]	0.12	0.10	0.285
	Natural log of total physical activity (MET-min/week)	−1.16 [−2.49; 0.17]	0.67	−0.16	0.087
	Weekday sitting time (min/week)	0.46 [−0.44; 1.37]	0.45	0.09	0.309
TBW (%)	Sex: Male (ref. Female)	5.82 [3.77; 7.87]	1.03	0.60	<0.001
	Age (years)	−0.48 [−0.79; −0.16]	0.16	−0.32	0.004
	Dance experience (years)	0.13 [−0.08; 0.34]	0.10	0.13	0.211
	Natural log of total physical activity (MET-min/week)	−1.03 [−2.19; 0.12]	0.58	−0.18	0.079
	Weekday sitting time (min/week)	0.37 [−0.42; 1.15]	0.39	0.09	0.358
Bone Mass (%)	Sex: Male (ref. Female)	0.37 [0.23; 0.51]	0.07	0.58	<0.001
	Age (years)	−0.02 [−0.04; 0.00]	0.01	−0.19	0.088
	Dance Experience (years)	0.01 [−0.01; 0.02]	0.01	0.11	0.276
	Natural log of total physical activity (MET-min/week)	−0.07 [−0.15; 0.01]	0.04	−0.19	0.069
	Weekday sitting time (min/week)	0.01 [−0.05; 0.06]	0.03	0.03	0.768
BMR (kcal/day)	Sex: Male (ref. Female)	545.66 [459.41; 631.92]	43.28	0.82	<0.001
	Age (years)	2.70 [−10.59; 15.99]	6.67	0.03	0.687
	Dance Experience (years)	−1.27 [−10.06; 7.52]	4.41	−0.02	0.774
	Natural log of total physical activity (MET-min/week)	67.16 [18.54; 115.78]	24.39	0.17	0.007
	Weekday sitting time (min/week)	16.99 [−16.08; 50.06]	16.59	0.06	0.309

B—unstandardized regression coefficient; SE—standard error; β—standardized regression coefficient; CI—95% confidence interval; *p*—*p* value. Female sex was used as the reference category. Dance experience was expressed in years. Total physical activity was natural-log transformed before inclusion in the models. For weekday sitting time, B, SE, and 95% CI are presented per 1000 min/week increase.

## Data Availability

Dataset available on reasonable request from the authors.
